# The HIV-1 Gp120/CXCR4 Axis Promotes CCR7 Ligand-Dependent CD4 T Cell Migration: CCR7 Homo- and CCR7/CXCR4 Hetero-Oligomer Formation as a Possible Mechanism for Up-Regulation of Functional CCR7

**DOI:** 10.1371/journal.pone.0117454

**Published:** 2015-02-17

**Authors:** Haruko Hayasaka, Daichi Kobayashi, Hiromi Yoshimura, Emi E. Nakayama, Tatsuo Shioda, Masayuki Miyasaka

**Affiliations:** 1 Laboratory of Immune Regulation, Department of Microbiology and Immunology, Graduate School of Medicine, WPI Immunology Frontier Research Center, Osaka University, Suita, Osaka, Japan; 2 Department of Viral Infections, Research Institute for Microbial Diseases, Osaka University, Suita, Osaka, Japan; 3 Institute for Academic Initiatives, Osaka University, Suita, Osaka, Japan; 4 MediCity Laboratory, University of Turku, Tykistökatu 6A, 20520, Turku, Finland; George Mason University, UNITED STATES

## Abstract

During human immunodeficiency virus (HIV) infection, enhanced migration of infected cells to lymph nodes leads to efficient propagation of HIV-1. The selective chemokine receptors, including CXCR4 and CCR7, may play a role in this process, yet the viral factors regulating chemokine-dependent T cell migration remain relatively unclear. The functional cooperation between the CXCR4 ligand chemokine CXCL12 and the CCR7 ligand chemokines CCL19 and CCL21 enhances CCR7-dependent T cell motility *in vitro* as well as cell trafficking into the lymph nodes *in vivo*. In this study, we report that a recombinant form of a viral CXCR4 ligand, X4-tropic HIV-1 gp120, enhanced the CD4 T cell response to CCR7 ligands in a manner dependent on CXCR4 and CD4, and that this effect was recapitulated by HIV-1 virions. HIV-1 gp120 significantly enhanced CCR7-dependent CD4 T cell migration from the footpad of mice to the draining lymph nodes in *in vivo* transfer experiments. We also demonstrated that CXCR4 expression is required for stable CCR7 expression on the CD4 T cell surface, whereas CXCR4 signaling facilitated CCR7 ligand binding to the cell surface and increased the level of CCR7 homo- as well as CXCR4/CCR7 hetero-oligomers without affecting CCR7 expression levels. Our findings indicate that HIV-evoked CXCR4 signaling promotes CCR7-dependent CD4 T cell migration by up-regulating CCR7 function, which is likely to be induced by increased formation of CCR7 homo- and CXCR4/CCR7 hetero-oligomers on the surface of CD4 T cells.

## Introduction

The human immunodeficiency virus type 1 (HIV-1) infects cells by utilizing its major envelope protein gp120 that binds to CD4 and also to chemokine receptors on human cells. In the case of CD4^+^ T cells, the HIV gp120 first binds to CD4 and then to CXCR4, which triggers fusion of viral and cellular membranes and confers virus entry to cells. The gp120/CD4/CXCR4 interaction also initiates various intracellular signaling pathways [1–4], which affect the migration patterns and activation status of target cells.

Under physiological conditions, recruitment of lymphocytes from the blood into the secondary lymphoid tissues is regulated by the interaction between lymphoid chemokines such as CCL19, CCL21, CXCL12, and CXCL13, and their specific G-protein-coupled receptors [5], [6]. CCL19 and CCL21 bind to a common receptor, CC-chemokine receptor 7 (CCR7) [7], [8], whereas CXCL12 acts on T and B cells through its specific receptor CXCR4 [9]. CXCL13 selectively interacts with CXCR5 in B cells [10], and mediates efficient B cell trafficking to Peyer’s patches and lymph nodes (LNs) [11], [12]. These lymphoid chemokines are selectively localized on the luminal surface and basal lamina of specialized venules of LNs known as high endothelial venules (HEVs), and in the parenchyma of the LNs and spleen [13], where they are presented to the circulating lymphocytes expressing corresponding G-protein-coupled receptors. The chemokine/chemokine receptor interaction induces β_2_ integrin activation, resulting in lymphocyte adhesion to HEV endothelial cells expressing selective adhesion molecules and subsequent cell migration across the HEV basal lamina [5], [6].

Although a single chemokine is able to bind to and activate its corresponding chemokine receptor(s), functional cooperation between different chemokines has also been reported in various cell types. CXCL13 promotes CCR7 ligand-dependent chemotaxis of peripheral blood lymphocytes [14], and CXCL12 and CCR5 ligand chemokines act cooperatively in chemokine-induced T cell costimulation [15]. It is also known that CXCR3 ligands [16] and CCR7 ligands act cooperatively with CXCL12 to enhance CXCR4 ligand-dependent plasmacytoid dendritic cell recruitment [17]. Previously, we reported that CXCL12 binding to CXCR4 enhanced CCR7 ligand-dependent chemotaxis and intracellular signaling events in T cells *in vitro* [18]. This enhancing effect of CXCL12 on CCR7 activity was also observed *in vivo*; CXCL12 promoted CCR7-dependent T cell binding to HEVs and their subsequent migration into the LN parenchyma [18]. Given that multiple lymphoid chemokines, including CXCL12 and CCR7 ligand chemokines, are closely localized on the luminal surface and/or the basal lamina of HEVs, it is tempting to speculate that the combinational effects of these chemokines might cause efficient and specific lymphocyte trafficking to the LNs and Peyer’s patches.

Green et al. [19] implies that non-chemokine ligands may also act to promote chemokine-induced T cell migration. In particular, they observed that the CXCR4-tropic (X4) envelope glycoprotein gp120 of HIV-1_IIIB_ promoted CD4 T cell responses to CCL21/CCR7 and CCL20/CCR6 as well as CD4 T cell accumulation in LNs. The promoting effect of HIV-1 on chemokine-dependent CD4 T cell migration raises the possibility that the gp120-CXCR4 interaction may enhance not only virus entry but also chemokine-dependent intracellular signaling and subsequent cell migration, thus contributing to rapid virus spread *in vivo*. However, the precise mechanism underlying the enhancing effects of gp120 on CCR7 and CCR6 responses as well as the actual involvement of CXCR4 or CCR7 in this process remain unclear.

In the current study, we examined the molecular mechanisms underlying the enhancing effect of gp120 on CCR7 responses using a recombinant HIV_NL4-3_-derived gp120. We determined that the enhancing effect was dependent on CXCR4 and CD4, and that not only recombinant gp120 but also the whole HIV virions enhanced CD4 T cell responses to CCR7 ligands. In addition, we found that CXCR4 signaling facilitated CCR7 ligand binding to the cell surface, thereby increasing the level of CCR7 homo- and CCR7/CXCR4 hetero-oligomers without affecting CCR7 expression levels. In the following sections, we will discuss the functional significance of gp120/CXCR4-induced CCR7 oligomer formation in the up-regulation of CCR7.

## Materials and Methods

### Antibodies and reagents

Functional-grade purified anti-human CXCR4 monoclonal antibody (mAb; clone 12G5) and anti-human CD4 mAb (RPA-T4) were purchased from eBioscience (San Diego, CA, USA). Anti-human CCR7 mAb (clone 150503), anti-human CCR1 mAb (clone 53504), recombinant human CXCL12/SDF-1α, recombinant human 6Ckine/CCL21, and recombinant soluble CD4 (sCD4) were purchased from R&D Systems (Minneapolis, MN, USA). The anti-HIV-1 gp120 mAb 902 was affinity purified from mouse ascites fluid. AMD3100 was purchased from Sigma-Aldrich (St. Louis, MO, USA). The virus-free and concentrated culture supernatant of CV1 cells infected with recombinant Sendai virus (SeV) expressing the envelope glycoprotein gp120 subunit derived from the HIV-1 X4 strain NL4-3, the virus-free and concentrated culture supernatant of CV1 cells infected with parental SeV, and affinity-purified HIV gp120 in elution buffer (80 mM acetic acid and 0.17 M Tris-HCl) were all prepared as previously described [20]. Wild-type and gp120-deficient HIV NL4-3 virions [21] were prepared from culture supernatants of the HIV-infected human T cell line MT4 and treated with aldrithiol-2 (AT-2) for 30 min at 37°C to inactivate their infectivity. Virus particles were concentrated through a 20% sucrose cushion by ultracentrifugation (120,000 × *g*, 2 h, 4°C) and resuspended in 10% fetal calf serum (FCS)/RPMI1640. Control samples were prepared from non-infected MT4 cells.

### Cells

Human peripheral blood mononuclear cells were obtained from healthy donors under written informed consent. Use of human materials in this study was approved by the Research Ethics Committee of Osaka University. Mononuclear cells were obtained by using Lymphoprep (Axis-Shield Diagnostics, Dundee, Scotland), according to manufacturer’s instructions, and CD4^+^ T cells were enriched using the CD4^+^ T Cell Isolation Kit II (Miltenyi Biotec, Bergisch Gladbach, Germany). A human CD4^+^ T cell line, H9 (HTB-176; obtained from American Type Culture Collection), was cultured in RPMI1640 supplemented with 10% (V/V) FCS, 2 mM l-glutamine, 1 mM sodium pyruvate, 100 U/ml penicillin, 100 μg/ml streptomycin, 50 μM 2-ME, 0.1 mM nonessential amino acids, and 10 mM HEPES.

### Cell migration assay

A time-lapse cell migration assay was performed using an EZ-TAXIScan system (GE Healthcare Japan, Tokyo, Japan) as reported previously [18]. CD4^+^ T cells (2 × 10^7^/ml) were resuspended in 0.1% fatty acid-free bovine serum albumin (BSA)/FCS-free RPMI1640 medium, and were pretreated with the gp120 culture supernatant, control culture supernatant, recombinant gp120 (1 μg/ml), or elution buffer for 30 min at room temperature in the presence of anti-CCR7 mAb, anti-CXCR4 antibody, control mouse IgG, sCD4, or AMD3100. After pretreatment, the cell suspension was loaded into each well of the EZ-TAXIScan microchamber. After cell alignment was complete, human CCL21 (100 ng) was applied to the contra-wells. Cell migration was recorded along a concentration gradient of the chemokine over a horizontal glass surface under a silicon chip with a CCD camera at time-lapse intervals, and the digital images were analyzed. The total number of migrated cells that reached the fixed window, which was located in the assay field two-thirds away from the starting point of cell migration, was counted, as described previously [22].

CD4 T cell chemotaxis was also analyzed by using a transwell-based double-chamber system with 3-μm pore-sized polycarbonate filters (ChemoTx) as described previously [18]. Briefly, purified human CD4^+^ T cells (1 × 10^5^ cells) were resuspended in 0.1% BSA/phenol red-free RPMI1640 medium, pretreated with the purified gp120 (5, 10, 20, or 40 μg/ml) or elution buffer for 30 min at room temperature, and were applied to the upper wells of a ChemoTx plate. CCL21 (250 or 1000 ng/ml) or vehicle control (phosphate-buffered saline, PBS) was applied to the lower wells. After 2 h incubation, the migrated cells were verified by the fluorometric determination of living cell numbers using a Cell Counting kit-F (Dojindo, Kumamoto, Japan). The fluorescence intensity was measured on a spectrofluorometer (SpectroMax; Molecular Devices, Sunnyvale, CA, USA).

### Animals

All animal experiments were performed using protocols approved by the Ethics Review Committee for Animal Experimentation of Osaka University Graduate School of Medicine (approved number: 24–103). C57BL/6 (Japan SLC, Hamamatsu, Japan) and *plt/plt* mice on the C57BL/6 background (provided by Dr. H. Nakano of the National Institute of Environmental Health Sciences, USA) were housed under specific pathogen-free conditions. All the injections were carried out under isoflurane anesthesia.

### Whole mount analysis

Human CD4 T cells were labeled with 10 μM 5-(and-6)-carboxyfluorescein diacetate, succinimidyl ester (CFSE; Invitrogen, Carlsbad, CA, USA) for 10 min at 37°C, and resuspended in RPMI1640 with 10% FCS. The labeled cells (5 × 10^6^ cells) were injected into the footpads of C57BL/6 or *plt/plt* mice. A sham operation (PBS injection) was performed on the contralateral side. Popliteal lymph nodes (pLNs) were harvested from recipient mice after the transfer and fixed with 4% paraformaldehyde, and then treated with 30% sucrose. The images of pLNs were analyzed by confocal microscopy (TCS SL or TCS SP5; Leica). The number of cells was counted by using the publicly available image analysis software Image J (National Institutes of Health, Bethesda, MD, USA).

### Flow-cytometric analysis

H9 cells (2 × 10^6^) were transfected with 20 pmol of CCR7, CXCR4, or control siRNA (SantaCruz, sc-39888, sc-35421, and sc-37007) using the Cell Line Nucleofector Kit R (Lonza, Basel, Switzerland), according to manufacturer’s instructions. Cells were harvested 10 hrs after transfection, and subjected to flow cytometry using anti-CCR7, anti-CXCR4, anti-CCR1 mAb, or control immunoglobulin. The fluorescence intensity of the AlexaFluor 488-conjugated goat anti-mouse IgG (Invitrogen) staining was measured by a FACSVerse (BD Biosciences) and analyzed by FlowJo software (Tree Star Inc., Palo Alto, CA). Detection of the CCR7 expression levels after gp120 or CXCL12 pre-treatment was perfomed as follows. Human peripheral mononuclear cells or H9 cells were preincubated with or without 0.1% BSA with PBS, CXCL12 (100 ng/ml), or recombinant gp120 (1 μg/ml) for 30 min, and then stained with phycoerythrin-conjugated anti-CCR7 mAb or control immunoglobulin. Human peripheral mononuclear cells were stained in the presence of with allophycocyanin-conjugated anti-human CD4 mAb for gating on the CD4^+^ cells. Each result shown is a representative result of three independent experiments.

### Western blotting

Cell lysates were prepared in 50 mM Tris-HCl, 20 mM NaF, 1 mM Na_3_VO_4_, 1% NaPO_4_, 2 mM EDTA, and 150 mM NaCl, with proteinase inhibitor cocktail (Sigma-Aldrich). The samples were separated by SDS-PAGE, transferred onto PVDF membranes for immunoblotting with a rabbit anti-CCR7 mAb (Abcam, Cambridge, MA, USA, clone Y59) and a biotin-coupled anti-rabbit IgG antibody (Merck Millipore, Darmstadt, Germany), and detected by using horseradish peroxidase (HRP)–conjugated streptavidin and the ECL system (GE Healthcare Japan). Anti-β actin mAb (Wako Pure Chemical Industries, Osaka, Japan, clone 2F3) and mouse anti-CCR7 mAb (clone 150503) were detected by a HRP-conjugated goat anti-mouse antibody (American Qualex, San Clemente, CA, USA).

### Ligand binding assay

H9 cells were seeded on fibronectin-coated wells and treated with 0.1% BSA with PBS, CXCL12 (100 ng/ml), or recombinant gp120 (1 μg/ml) for 30 min. Binding of CCL19-Ig (1 μg/ml; eBioscience) to T cells was detected by staining with 5 μg/ml biotin-conjugated anti-human IgG (American Qualex) and 5 μg/ml Alexa Fluor 647-conjugated streptavidin (Invitrogen). The images were captured by a confocal microscope and analyzed with Duolink Image Tool software (Olink Bioscience, Uppsala, Sweden).

### Proximity ligation assay

CCR7 homo-oligomer formation was examined by the Duolink proximity ligation assay (PLA; Olink Bioscience), in which oligomerized CCR7 appears as orange dots representing the presence of a protein-protein interaction complex. Briefly, H9 cells in culture media containing 0.1% BSA were treated with or without CXCL12 or recombinant gp120 in fibronectin-coated wells for 30 min, fixed with 4% paraformaldehyde, and stained with a 7.5 μg/ml anti-human CCR7 mAb (R&D, MAB197) independently conjugated with the complementary oligonucleotide probe generated by Duolink II Probemaker PLUS or MINUS (Olink Bioscience). CCR7/CXCR4 hetero-oligomerization was examined by using anti-human CCR7 and anti-human CXCR4 mAbs (eBioscience, clone 12G5), which were labeled with the PLA-Minus and the PLA-Plus probes, respectively. As a control, anti-human CCR1 mAb (R&D, clone 53504) was used. The complementary DNA strands in close proximity (<40 nm) were amplified using fluorescence, which were visualized using a confocal laser microscope. The images were analyzed using the Duolink Image Tool software (Olink Bioscience).

## Results

### *The recombinant X4-tropic gp120* and a CCR7 ligand chemokine act synergistically on CD4 T cell migration

**Fig 1:**
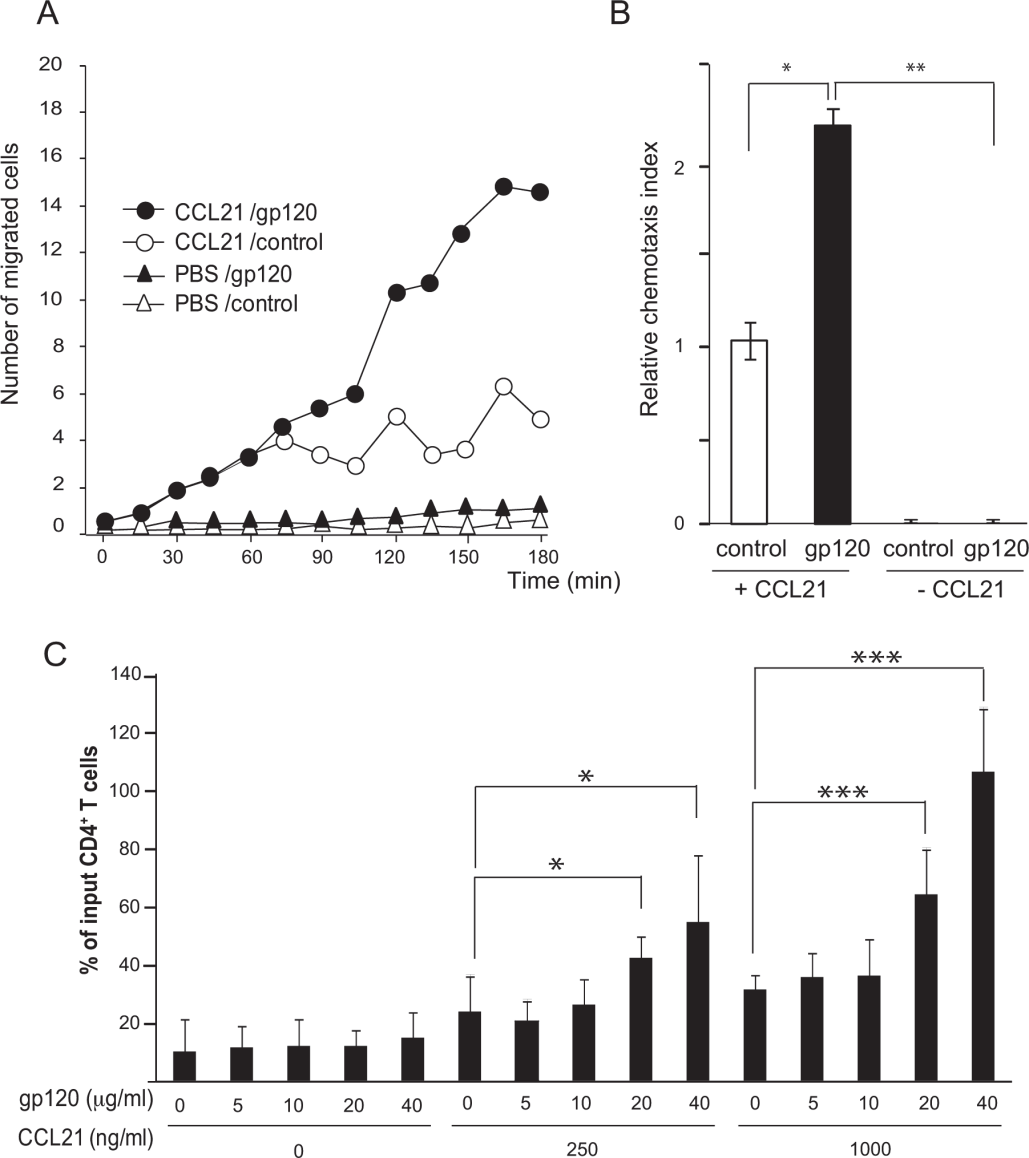
HIV-1_NL4-3_-derived gp120 promoted CCL21-induced CD4 T cell chemotaxis. (A) Human CD4 T cells were incubated for 30 min at room temperature with the culture supernatant containing the recombinant HIV-1_NL4-3_-derived gp120 (50 μg/ml, 420 nM) or a control solution, and chemotaxis in response to CCL21 was analyzed on a time-lapse video microscope. The number of cells in the fixed window, which was located in the assay field two-thirds away from the starting point of cell migration, was plotted against time after CCL21 injection. The result shown is a representative result of three independent experiments. (B) Quantification of the CCL21-induced CD4 T cell chemotaxis shown in panel A. The total number of migrated cells was calculated by summing the individual cell counts. The relative chemotaxis index represents the ratio of the number of migrated cells in the test treatment to that in control treatments (control solution in the presence of CCL21). Data represent means ± SEM of three independent experiments. *, p < 0.05 **, p < 0.01. (C) Human CD4 T cells (1 × 10^5^) were mixed with the purified gp120 or control elution buffer and added to the upper wells of the transwell plate. The number of migrated cells in response to CCL21, which was added to the lower wells of the transwell plate, was analyzed. Graphs represent means ± SD percentage of input cells from triplicate wells. *, p < 0.05 ***, p < 0.001 by the Student’s *t*-test.

To examine the effect of recombinant gp120 on CCR7-dependent CD4 T cell migration, we evaluated the number of migrated T cells in response to CCL21 in the presence or absence of the recombinant HIV_NL4-3_-derived gp120 in a real-time micro-chemotaxis chamber. CD4 T cells resuspended in the recombinant gp120-containing cell culture supernatant showed more efficient migration in the direction of the CCL21 gradient compared with those in the control (gp120-free) supernatant (Fig. 1A). By the end of the 3-h experimental period, the number of migrated CD4 T cells in the gp120 pretreatment group was approximately double that in the control treatment group (Fig. 1B). However, in the absence of CCL21, gp120 did not affect the number of migrating cells, indicating that CD4 T cell migration is largely dependent on CCL21 (Fig. 1B). These results support the previous study by Green et al. [19] who showed that X4-tropic HIV-1_IIIB_-derived gp120 enhances the responsiveness of CD4 T cells to CCR7 ligand chemokines.

We next examined the effect of purified recombinant HIV-1 gp120 on CD4 T cell migration in a transwell-based chamber assay (Fig. 1C). HIV-1 gp120 alone did not induce CD4 T cell migration at the concentrations examined in the absence of CCL21. In contrast, at concentrations of 20 μg/ml or higher, gp120 enhanced CD4 T cell migration at least 2-fold over the basal levels when 250 ng/ml or 1,000 ng/ml CCL21 was applied to the lower wells of the chamber. The enhancing effect of gp120 was dose-dependent, and was more prominent with an increase in CCL21 concentration, suggesting that gp120 and CCL21 cooperate synergistically on CD4 T cell migration.

### HIV-1 gp120 promotes CCL21-induced CD4 T cell chemotaxis in a CXCR4- and CD4-dependent manner

**Fig 2:**
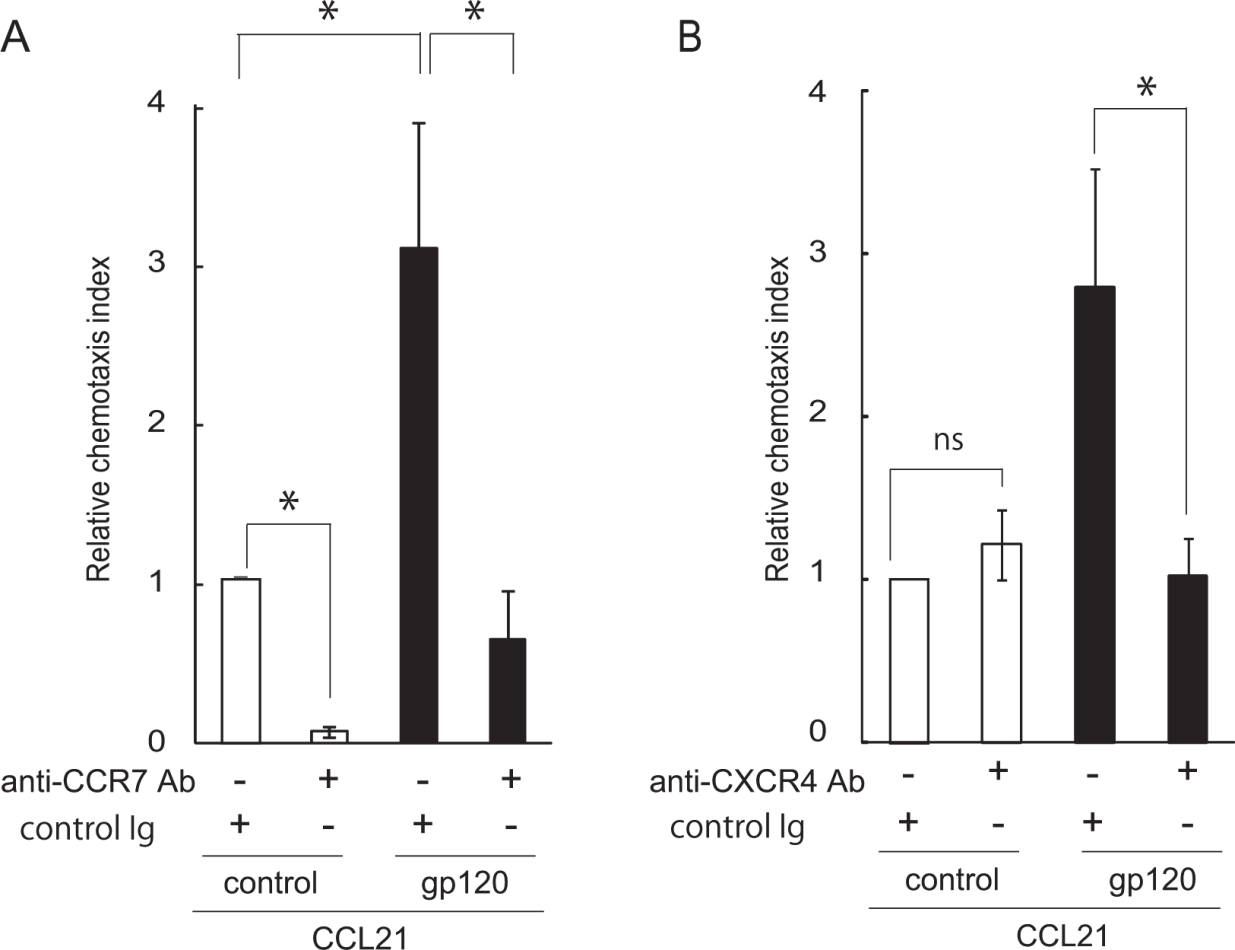
The effect of gp120 on CCL21-induced chemotaxis was dependent on CCR7 and CXCR4. (A) Human CD4 T cells were incubated for 30 min at room temperature with the culture supernatant containing recombinant gp120 (50 μg/ml) or a control solution, and chemotaxis in response to CCL21 was analyzed on a time-lapse video microscope. The total number of migrated cells was calculated by summing the individual cell counts. The assay was performed in the presence of a neutralizing anti-CCR7 antibody or control IgG (25 μg/ml). The relative chemotaxis index represents the ratio of the number of migrated cells in the test treatment to that in control treatments (control solution and control IgG). Data represent means ± SEM of three independent experiments. *, p < 0.05 by the Student’s *t*-test. (B) The assay described above was performed in the presence of a neutralizing anti-CXCR4 antibody or control IgG. The relative chemotaxis index represents the ratio of the number of migrated cells in the test treatment to that in control treatments (control solution and control IgG).

To understand the mechanism underlying the enhancing effects of gp120 on CCR7 ligand-mediated responses, we examined its CXCR4 dependency. As expected, the number of migrated cells in response to CCL21 was significantly increased in the presence of HIV-1 gp120 and reduced to the basal level following application of a neutralizing anti-CCR7 antibody (Fig. 2A), confirming its CCR7 dependency. In addition, CD4 T cell migration was also almost completely reduced to the basal level in the presence of an anti-CXCR4 neutralizing antibody (Fig. 2B) although the number of migrated cells was not altered in response to treatment of CCL21 alone, indicating that gp120 acts primarily through CXCR4 to enhance CCR7-dependent CD4 T cell chemotaxis.

**Fig 3:**
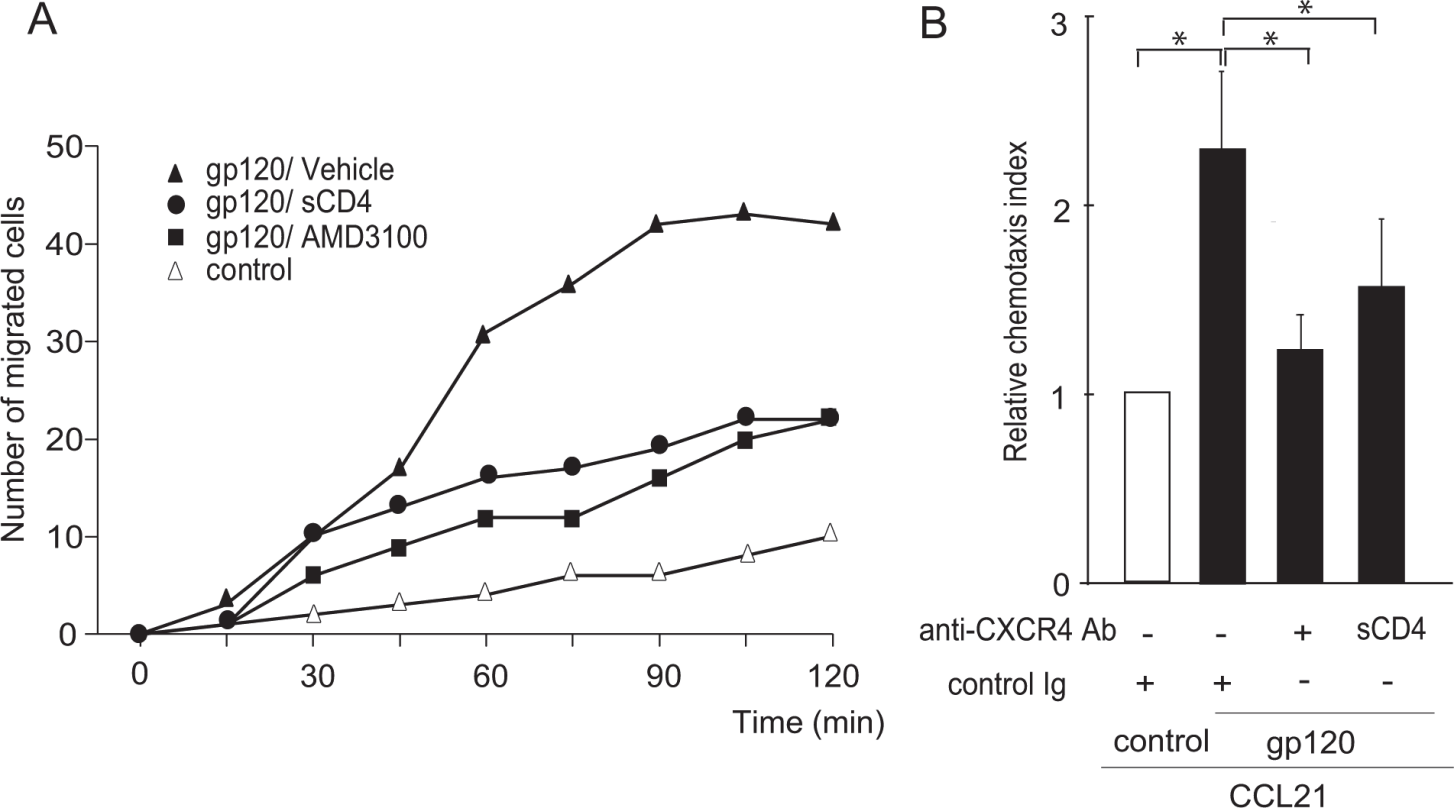
HIV-1 gp120 acted on CXCR4 and CD4 to promote CCR7-dependent human CD4 T cell migration. (A) CCR7 ligand-induced H9 cell migration after treatment with 1 μg/ml recombinant gp120 (filled symbols) or control buffer (open triangles) was examined on a time-lapse video microscope. CCL19-induced cell migration in H9 cells was examined in the presence of AMD3100 (25 μg/ml, filled squares), sCD4 (3 μg/ml, filled circles), or vehicle (filled triangles). The result shown is a representative result of three independent experiments. (B) CCR7 ligand-induced cell migration in the presence of a neutralizing anti-CXCR4 antibody, sCD4, or control IgG in H9 cells after treatment with 1 μg/ml recombinant gp120 (black bars) or control buffer (open bars). CCL21-induced cell migration was examined by a time-lapse video microscope, and the total number of migrated cells was calculated. The relative chemotaxis index represents the ratio of the number of migrated cells in the test treatment to that in control treatments (control solution in the presence of IgG). Data represent means ± SEM of three independent experiments. *, p < 0.05 by the Student’s *t*-test.

It has been well established that HIV-1 gp120 forms a trimolecular complex with CD4 and CXCR4 in CD4 T cells [23]. We thus investigated the effect of a CXCR4 antagonist, AMD3100, and sCD4 on CCL21-induced migration using a CD4- and CXCR4-expressing H9 human T-lymphoma cell line; this cell line showed enhanced CCL21- or CCL19-dependent chemotaxis in the presence of gp120 at a comparable level to that observed in primary human CD4 T cells (data not shown). As shown in Fig. 3A, the enhancing effect of gp120 on CCL19-induced chemotaxis was significantly inhibited by AMD3100 or sCD4, as assessed in a real-time micro-chemotaxis chamber assay. In addition, in a transwell-based chamber assay, the effect of gp120 was inhibited, with cell numbers reaching almost basal levels, by a neutralizing anti-CXCR4 antibody, and was partially inhibited by sCD4 (Fig. 3B), confirming its dependency on CXCR4 and CD4.

### HIV-1 virions promote CCR7-induced CD4 T cell migration

**Fig 4:**
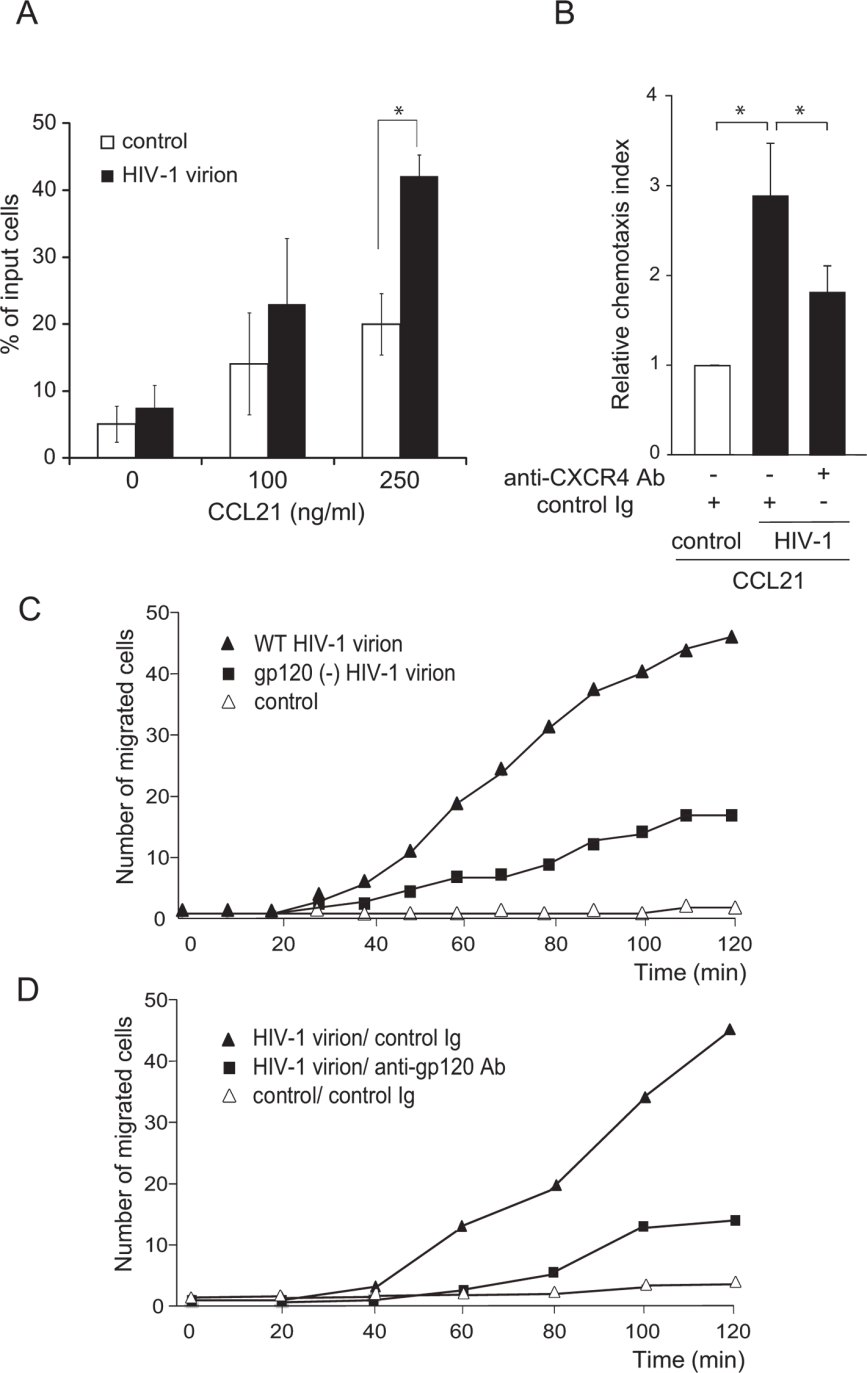
HIV-1 virions enhanced CCL21-induced CD4 T cell chemotaxis. (A) A CCR7 ligand-induced cell migration assay was performed using H9 cells (5 × 10^4^) pre-treated with or without AT-2-inactivated HIV-1 virions (final concentration corresponding to 10 μg/ml p24) or control supernatant for 15 min. The number of migrated cells in response to CCL21 added to the lower wells of the transwell plate was analyzed. Graphs represent means ± SD percentage of input cells from triplicate wells. *, p < 0.05 by the Student’s *t*-test. The result shown is a representative result of three independent experiments. (B) The time-lapse cell migration assay was performed in the presence of a neutralizing anti-CXCR4 antibody or control IgG. The relative chemotaxis index represents the ratio of the number of migrated cells in the test treatment to that in control treatments (control AT-2-treated mock supernatant in the presence of IgG). Data represent means ± SEM of three independent experiments. *, p < 0.05 by the Student’s *t*-test. (C) CCR7 ligand-induced H9 cell migration after treatment with AT-2-inactivated wild-type (WT) HIV-1 (filled triangles), gp120-deficient HIV-1 virions (filled squares) or control mock supernatant (open triangles). CCL19-induced cell migration was examined on a time-lapse video microscope. The result shown is a representative result of three independent experiments. (D) CCR7 ligand-induced H9 cell migration after treatment with AT-2-inactivated HIV-1 virions (filled symbols) or control supernatant (open triangles) in the presence of a neutralizing anti-gp120 antibody (50 μg/ml, filled squares) or control IgG (filled triangles). CCL21-induced cell migration was analyzed on a time-lapse video microscope. The result shown is a representative result of three independent experiments.

We next examined whether the native gp120 expressed on the viral envelope behaves in the same manner as recombinant gp120. As shown in Fig. 4A, whereas HIV-1 virion-containing culture media hardly affected the number of migrated cells in the absence of CCL21, cell migration was enhanced in the presence of CCL21 (250 ng/ml). A 2-fold increase in the number of migrated cells was observed in HIV-1 virion media compared with HIV-1-free culture media (Fig. 4A), and the enhancing effect was significantly inhibited in the presence of a neutralizing anti-CXCR4 antibody (Fig. 4B). The enhancing effect was significantly, albeit not completely, abrogated in a mutant HIV-1 lacking gp120 (Fig. 4C), indicating that at least a part of HIV-1 virion’s effect is gp120-dependent. Supporting this result, the enhancing effect of HIV-1 virion was significantly inhibited by anti-gp120 neutralizing antibody (Fig. 4D). These results indicate that gp120 on the HIV viral envelope promotes CCR7-dependent CD4 T cell migration in a manner dependent on CXCR4.

### HIV-1 gp120 promotes CCR7-dependent CD4 T cell trafficking to lymph nodes

**Fig 5:**
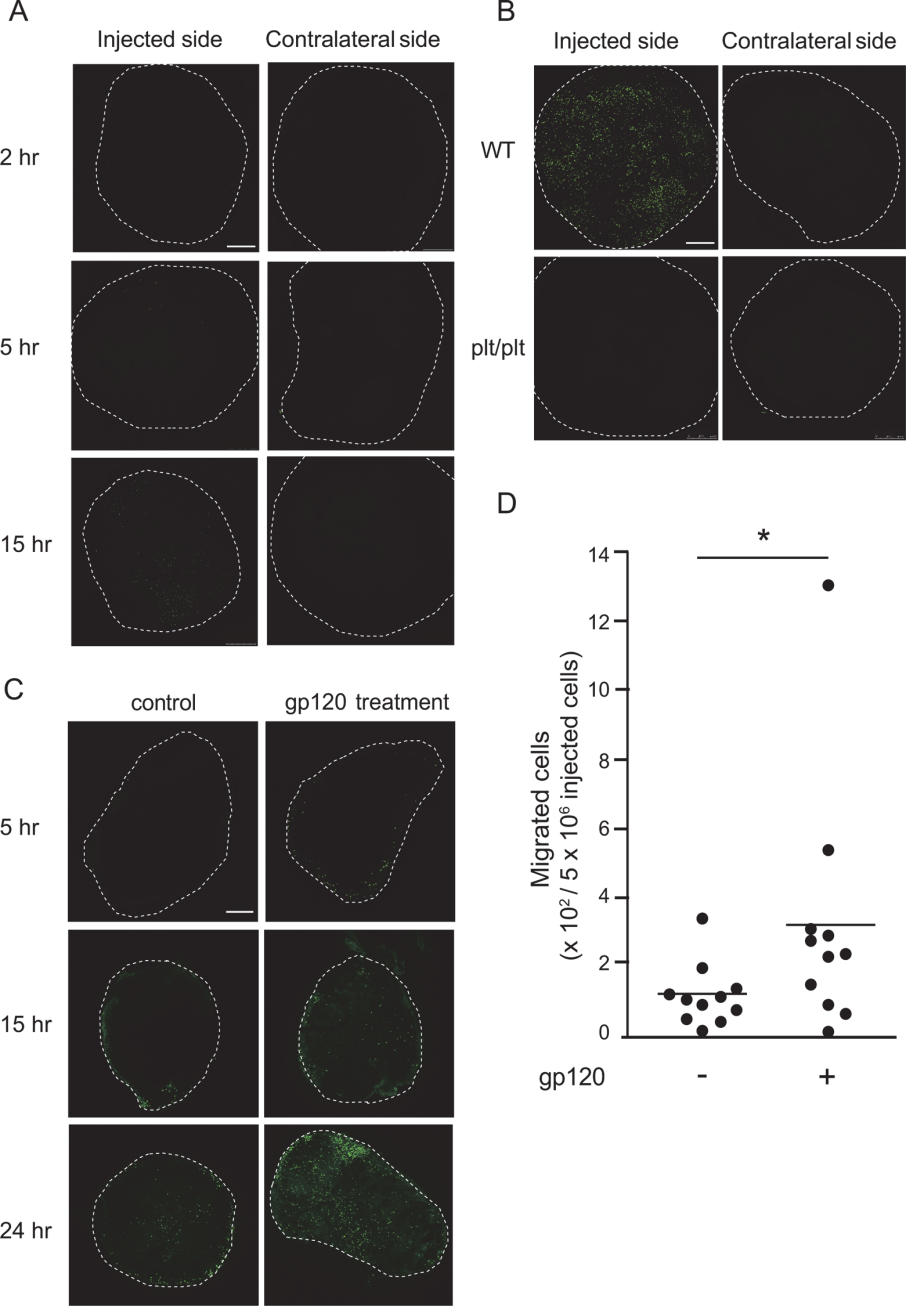
HIV-1 gp120 promoted CCR7-dependent CD4 T cell trafficking to the popliteal lymph nodes. (A) CFSE-labeled human CD4 T cells (5 × 10^6^) were injected into the footpads of C57BL/6 WT mice. A sham operation (PBS injection) was performed on the contralateral side. Popliteal lymph nodes (pLNs) were harvested from the recipient mice at 2 h, 5 h, and 15 h after the transfer. (B) CFSE-labeled human CD4 T cells (5 × 10^6^) were injected into the footpads of C57BL/6 WT mice or *plt/plt* mice. pLNs were harvested from the recipient mice at 15 h after cell transfer. The images of pLNs were analyzed by fluorescence confocal microscopy (original magnification ×100). Scale bar, 250 μm. (C) CFSE-labeled human CD4 T cells (5 × 10^6^) were pretreated with purified gp120 (40 μg/ml) or control elution buffer, and injected into each side of C57BL/6 mouse footpad. pLNs were harvested from recipient mice at 5, 15, and 24 h after the transfer and analyzed by confocal microscopy (TCS SP5; Leica). Representative images of pLNs obtained from 11 recipient mice subjected to CD4 T cell injection are shown. (D) The images obtained from individual mice (n = 11) at 5 h after the transfer were analyzed and the total numbers of migrated cells per 5 × 10^6^ cells in a recipient mouse tissue was quantified using ImageJ software (NIH). *, p < 0.05 by the Wilcoxon signed-rank test.

Previously, Green et al. [19] reported that treatment of lymphocytes with gp120 from X4-tropic HIV_IIIB_ enhanced blood-borne lymphocyte migration to the LNs. In the present study, we investigated whether gp120 from X4-tropic HIV_NL4-3_ promotes lymph-borne T cell trafficking into lymphoid tissues in a manner dependent on CCR7. Given that lymphatic migration of T cells from the footpad to draining pLNs is CCR7-dependent and can be quantified by counting the number of migrating cells from the footpad to the pLN in the mouse [24], we first tested whether human T cells would migrate appropriately upon footpad injection in the mouse. After 2 h, injected T cells were observed in the pLN draining the injection side and their numbers gradually increased in a time-dependent manner (Fig. 5A), indicating that human T cells successfully migrated to the LNs via the lymphatic system of the mouse. At 15 h post injection, a large number of labeled CD4 T cells were observed in the pLN ipsilateral to the injected side but not in the contralateral pLN, confirming that mouse cells migrated into pLNs via the lymphatics but not via the blood vessels (comparable numbers of labeled cells would have appeared in ipsi- and contralateral pLNs if they had migrated via the blood vessels). Strikingly, when CCR7 ligand-deficient *plt/plt* mice were used as recipients, hardly any donor T cells appeared in the pLN even 15 h after injection (Fig. 5B), which further supports the hypothesis that their migration is CCR7-dependent. We then examined the effect of gp120. When human CD4 T cells were pretreated with purified recombinant gp120, they accumulated more efficiently in the pLN than those treated with control solution at time points examined (5, 15, 24 h) after footpad injection (Fig. 5C). The total number of migrated cells to the pLN was significantly higher in the gp120-treated group than that in the control group (Fig. 5D). In contrast, gp120-treated cell hardly migrated to the pLN *plt/plt* mice (data not shown). Collectively, these data support the notion that HIV gp120, in either the recombinant form or the native form expressed on the viral envelope, promotes lymphatic migration of CD4 T cells into peripheral LNs in a CCR7-dependent manner.

### CXCR4 is required for stable CCR7 expression, and CXCR4 signaling promotes CCR7 ligand binding without affecting CCR7 expression

**Fig 6:**
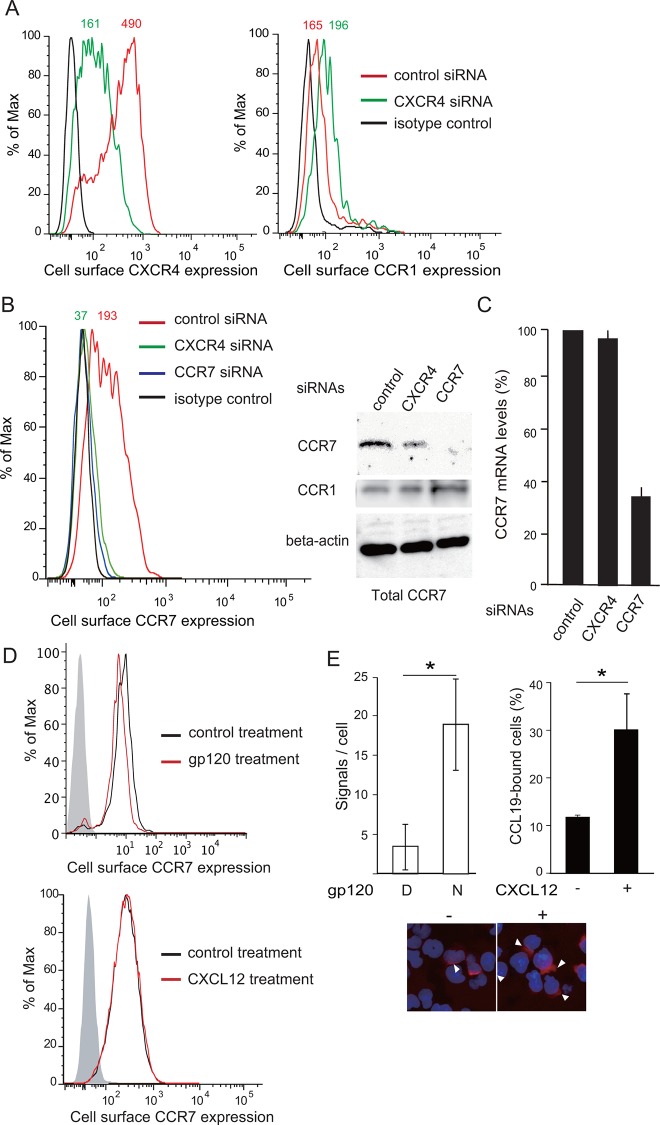
CXCR4 is required for stable CCR7 expression, CCR7 ligand binding, and CCR7 homo-oligomer formation. (A) The cell surface expression levels of CXCR4 and CCR1 were evaluated by flow cytometry using anti-CXCR4 (left) or anti-CCR1 (right) antibodies in control or CXCR4 siRNA-treated H9 cells. Mean fluorescence intensity is indicated on the histograms. (B) The cell surface CCR7 expression was evaluated by flow cytometry using anti-CCR7 antibody. H9 cells treated with control, CXCR4, or CCR7 siRNAs were analyzed (left). The total cellular CCR7 and CCR1 expression levels in control, CXCR4, or CCR7 siRNA-treated H9 cells were analyzed by Western blotting. Anti-β actin mAb was used to confirm equal loading (right). (C) The *CCR7* mRNA expression levels were analyzed in control, CXCR4, or CCR7 siRNA-treated H9 cells by quantitative RT-PCR. (D) The cell surface CCR7 expression levels were analyzed in gp120-treated human CD4 T cells (upper panel), and CXCL12-treated H9 cells (lower panel) by flow cytometry. (E) The CCR7 ligand-binding abilities in H9 cells pretreated with a native (N) or control, heat-denatured gp120 (D), or with (+) or without (−) 100 ng/ml CXCL12 were examined by CCL19-Ig binding. A minimum of three images per section was observed by confocal microscopy, and the relative fluorescence signal or the percentage of CCL19-Ig bound cells was quantified using Duolink Image Tool software. The images of CCL19-bound cells with or without CXCL12 pretreatment are shown in the right panel (indicated by white triangles). *, p < 0.05, Student’s *t*-test.

We next sought to determine the molecular mechanism by which CXCR4 signaling promotes CCR7-dependent CD4 T cell migration. To this end, we employed a small interfering RNA (siRNA) approach to suppress the cell surface expression of CXCR4 in CD4 T cells. We observed that CXCR4 siRNA selectively reduced CXCR4 expression by approximately three-fold (Fig. 6A, left). Interestingly, however, CXCR4 siRNA significantly diminished both the cell surface and total CCR7 expression in H9 cells (Fig. 6B). In contrast, CXCR4 siRNA did not reduce but rather slightly increased the cell surface CCR1 expression (Fig. 6A, right). This occurred without any changes in CCR7 expression at the mRNA level (Fig. 6C), supporting the hypothesis that CXCR4 expression contributes to stable CCR7 expression.

We then examined whether CXCR4 signaling affects CCR7 quantitatively or qualitatively. Preliminary experiments indicated that treatment with recombinant gp120 or CXCL12, at any of the concentrations that showed enhancing effects, did not alter CCR7 expression levels in human peripheral blood CD4 T cells and H9 cells (Fig. 6D). This result suggests that the enhancing effects of CXCR4 ligands on CCR7 signaling are not mediated by quantitative changes in CCR7 expression. We thus evaluated the ligand binding ability of CCR7 before and after CXCR4 ligation with CXCL12 or HIV gp120. As shown in Fig. 6E, gp120 or CXCL12 treatment increased the level of CCL19-Ig fusion protein binding to CD4 T cells by 3–5 times compared with mock treatment, in agreement with the hypothesis that CXCR4 signaling facilitates CCR7 ligand binding without affecting CCR7 expression levels, thus enhancing CCR7-mediated responses in CD4 T cells.

### CXCR4 signaling promotes CCR7 homo- and CXCR4/CCR7 hetero-oligomerization

**Fig 7:**
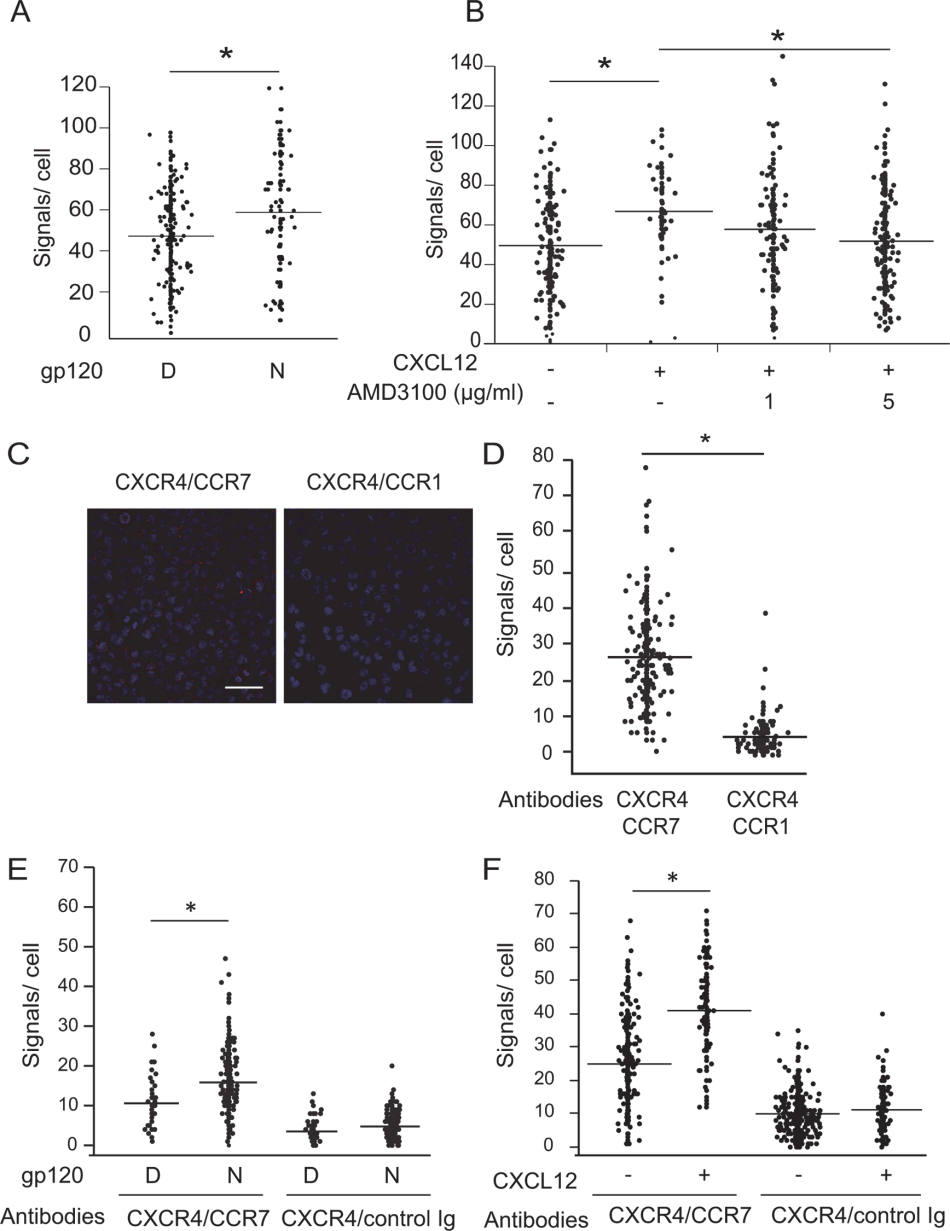
CXCR4 ligand binding facilitates CXCR4/CCR7 hetero-oligomer formation. (A) CCR7 homo-oligomer formation in H9 cells after treatment with or without 1 μg/ml recombinant gp120 was examined by *in situ* PLA. The number of *in situ* PLA signals per cell was counted by using the Duolink Image Tool software. The result shown is a representative result from three independent experiments showing the mean number of the signals plotted on the vertical axis. *, p < 0.05 by Mann-Whitney’s U test. (B) CCR7 homo-oligomer formation in H9 cells after treatment with 100 ng/ml CXCL12 was examined, as described in (A). Quantification of CCR7 homo-oligomers after CXCL12 pretreatment in the presence or absence of 1 or 5 μg/ml AMD3100. The result shown is a representative result from five independent experiments. *, p < 0.05 by Mann-Whitney’s U test. (C) CXCR4/CCR7 or CXCR4/CCR1 hetero-oligomer formation in H9 cells by *in situ* PLA using the indicated combinations of antibodies. The z-stack images derived from sections covering 10 μm with a 0.5-μm step are shown. Scale bar represents 50 μm. (D) Quantification of the detected *in situ* PLA signals per cell was performed. The result shown is a representative result from three independent experiments. *, p < 0.05 by Mann-Whitney’s U test. (E) CXCR4/CCR7 hetero-oligomer formation in H9 cells after treatment with 1 μg/ml gp120 in a native form (N) or control heat-denatured form (D) was examined by the PLA using the indicated combinations of antibodies. (F) CXCR4/CCR7 hetero-oligomer formation was examined with 100 ng/ml CXCL12 as described in (E).

As receptor oligomerization is an important process for activating chemokine receptors [25], we next examined whether CXCR4 ligand binding affects CCR7 oligomerization on the surface of CD4 T cells. Using the *in situ* DuoLink PLA [26], we found that native gp120 significantly increased the signals generated by CCR7 homo-oligomers compared with a heat-denatured gp120 in CD4 T cells (Fig. 7A). Similarly to gp120, CXCL12 also increased CCR7 homo-oligomerization signals, and CXCL12’s enhancing effect was inhibited by addition of a CXCR4 inhibitor, AMD3100, in a dose-dependent manner (Fig. 7B), indicating that the CXCL12-induced CCR7 homo-oligomerization is CXCR4-dependent.

Interestingly, CCR7 seemed to associate with not only CCR7 but also CXCR4, which appears to take place under constitutive conditions in CD4 T cells. Using the PLA, strong signals derived from CXCR4/CCR7 hetero-oligomers were observed even in the absence of CXCR4 or CCR7 ligands, whereas only very low signals were observed with CXCR4/CCR1 oligomers under the same conditions (Fig. 7C, D); CCR1 is a chemokine receptor expressed at a level comparable to that of CCR7 in H9 cells. These results indicate a basal association of CXCR4 with CCR7 on the surface of CD4 T cells. The basal CXCR4/CCR7 association appears to increase upon HIV gp120 binding to CXCR4. As shown in Fig. 7E, native HIV-1 gp120, but not its heat-denatured form, increased the level of CXCR4/CCR7 hetero-oligomerization by approximately two-fold. CXCL12 also showed an enhancing effect on CXCR4/CCR7 hetero-oligomerization (Fig. 7F). These results suggest that CXCR4 ligand-induced signaling promotes CXCR4/CCR7 hetero-oligomerization in CD4 T cells.

## Discussion

### CXCR4 binding molecules stimulate CCR7-dependent CD4 T cell responses

In this study, we demonstrated that the recombinant X4-HIV-1_NL4-3_-derived gp120 enabled human CD4 T cells to respond to minimum concentrations of CCL21 or CCL19, suggesting that HIV-1 gp120 cooperated with CCR7 ligands to enhance CD4 T cell migration. Our results are partly consistent with those reported by Green et al. [19], who demonstrated that X4-HIV-1_IIIB_-derived gp120 promoted CCL21-dependent CD4 T cell migration. However, whereas they found that gp120 cooperates with CCL21 but not with another CCR7 ligand, CCL19, we observed effective cooperation for both CCR7 ligands. This discrepancy may be due to the different sources of gp120 preparation between studies, which may influence the affinity of gp120 for CXCR4.

### Requirement of CXCR4 and CD4 for the enhancing effect of gp120

As the X4 gp120 can interact directly with CXCR4 independently of CD4 [27], [28] and mimics the effects of CXCL12 in cellular responses via CXCR4 [3], [4], [29], we speculated that gp120 directly exerts its enhancing effects via CXCR4. Accordingly, we found that gp120’s promoting effect was inhibited almost to the basal level in the presence of a neutralizing anti-CXCR4 antibody *in vitro* (Fig. 2B), whereas the effect of HIV-1 gp120 was inhibited by sCD4 to a lesser extent. This result supports the idea that X4 gp120 acts primarily through CXCR4 to promote CCR7-dependent cell migration and that the interaction between gp120 and CD4 may be less important in this process. However, considering that HIV-1 gp120 activates multiple CD4-mediated intracellular signaling pathways, such as T cell apoptosis [30], mitogen-activated protein kinase pathways [2], and chemokine receptor down-regulation [31], [32], gp120 binding to CD4 may also play a role in enhancement of CCR7-dependent T cell trafficking *in vivo*.

### A possible mechanism of CXCR4-mediated CCR7 sensitization

Our current and previous data [18] have shown that neither CXCL12 nor gp120 significantly increased cell surface CCR7 expression, suggesting that CXCR4 signaling increases CCR7 ligand binding through a mechanism other than the quantitative change in CCR7 receptor expression on the T cell surface. Recently, several reports have shown that oligomerization of chemokine receptors is involved in their functional activation, ligand binding, and induction of specific signaling cascades [25]. We found that CCR7 constitutively formed homo-oligomers at basal levels in the absence of CCR7 or CXCR4 ligands, and that this homo-oligomerization was significantly enhanced after CXCR4 signaling. We also found that CCR7 constitutively forms heterodimers with CXCR4, which was promoted subsequent to CXCR4 ligand stimulation. The observed formation of homo- and hetero-oligomers raises questions as to whether these complexes play a functional role in the up-regulation of CCR7-dependent signaling by CXCR4 ligands. We speculate that induction of CCR7 homo-oligomerization is coupled to direct receptor activation, as some chemokine receptors are activated through receptor dimerization upon ligand binding; for example, CXCL12-induced CXCR4 dimerization activates CXCR4’s downstream signaling pathway [33]. In addition, ligand-dependent CCR2 dimerization was observed with the monocyte chemoattractant protein-1 receptor, CCR2, and this dimerization was shown to be functionally relevant in the ligand-induced signal transduction pathway [34]. It is also likely that CXCR4/CCR7 hetero-oligomerization may directly and/or indirectly modulate CCR7 responses; that is, ligand binding to CXCR4 may influence the conformational state, ligand binding affinity, and assembly of signaling complexes to CCR7. Indeed, there are several reports demonstrating the importance of chemokine receptor cross-communication for signal amplification and diversification; CCR2/CCR5 hetero-oligomerization increases the receptor sensitivity to chemokine ligands and triggers distinct signaling pathways [35]. CXCR7/CXCR4 heterodimerization has been shown to alter CXCR4-mediated G-protein-coupled signaling [36] and preferentially activates the alternative β-arrestin-linked signaling pathway [37]. In addition, we speculate that ligand-independent CXCR4/CCR7 oligomerization may contribute to maintaining the functional CCR7 level, which is supported by the results of our siRNA knockdown experiments showing that CXCR4 contributes to stable CCR7 expression on the plasma membrane. To verify the involvement of these receptor oligomerizations in regulating CCR7-ligand responsiveness, we are currently adopting an experimental approach previously reported [38], [39] to disrupt CXCR4 or CCR5 oligomers with synthetic peptides that can destabilize chemokine receptor oligomerization.

### Possible contribution of gp120 to CD4 T cell trafficking *in vivo*

Whereas Green et al. [19] also demonstrated that gp120-sensitized lymphocytes migrated to LNs in a CCR7-dependent manner, this result was observed following intravenous injection of lymphocytes in immunocompromised NOD-SCID mice. In contrast, we demonstrated the gp120-induced promotion of CD4 T cell migration upon injection of lymphocytes into the footpads of normal mice, because the lymphatics are considered to be an important route for the systemic spread of HIV-infected T cells [40] [41]. Thus, we suggest that HIV gp120 contributes to both the hematogenous and lymphatic spread of CD4 T cells. These data indicate that inhibition of gp120 activity may help to normalize T cell trafficking, which may in turn help to restore immunocompetence in HIV-infected patients.

It has been reported previously that CD4 T cells are sequestered in lymphoid tissues and then reappear in the blood after active antiretroviral therapy in HIV-infected patients [42], implying that the CD4 T cell distribution can be regulated by certain signals from the virus itself. Although the mechanistic basis for this phenomenon remains unclear, our findings that gp120 promotes CCR7-dependent T cell migration in a CXCR4-dependent manner shed some light in this regard.

In our previous study using mouse T cells, CXCL12 concentrations of 250 nM or higher were required to observe a synergistic effect on CCR7-dependent migration *in vitro* [18], whereas in this study, HIV gp120 concentrations of only 170 nM or greater were required to observe the enhancing effect on primary human CD4 T cells. From these results, we speculate that nanomolar concentrations of CXCR4-binding molecules would be required for generating a synergistic effect on CCR7-dependent cell migration. Although there is currently no accurate information related to the CXCL12 and gp120 concentrations in local tissues, the reported plasma level of CXCL12 is ~200 pM [43] and the concentrations of gp120 in HIV-infected patients’ sera vary from 2 to 800 pM depending on sample preparation and quantifying methods [44], which are much lower levels than those used in our *in vitro* experiments. On the other hand, the CXCR4 ligand concentrations in specific tissue microenvironments are speculated to be in the micromolar range because CXCL12 strongly binds to tissue matrix components, including glycosaminoglycans/proteoglycans [45], and gp120 is also captured by matrix components [46] or dendritic cells [47]. In addition, the finding that CXCL12 functions more effectively when in immobilized form than soluble form [48] supports the idea that CXCR4 ligands can be presented at functional concentrations to promote CCR7-dependent T cell migration *in vivo*. The specific biological role of soluble and/or matrix-associated CXCR4 ligands in controlling CCR7-dependent CD4 T cell trafficking is an important topic for further research in understanding the role of CD4 T cell migration in HIV-1 pathogenesis.
